# Dance and Music in “Gangnam Style”: How Dance Observation Affects Meter Perception

**DOI:** 10.1371/journal.pone.0134725

**Published:** 2015-08-26

**Authors:** Kyung Myun Lee, Karen Chan Barrett, Yeonhwa Kim, Yeoeun Lim, Kyogu Lee

**Affiliations:** 1 Smart Humanity Convergence Center, Graduate School of Convergence Science and Technology, Seoul National University, Seoul, Korea; 2 Peabody Institute of Music at Johns Hopkins University, Baltimore, Maryland, United States of America; 3 Graduate School of Convergence Science and Technology, Seoul National University, Seoul, Korea; 4 College of Music, Seoul National University, Seoul, Korea; University of Bath, UNITED KINGDOM

## Abstract

Dance and music often co-occur as evidenced when viewing choreographed dances or singers moving while performing. This study investigated how the viewing of dance motions shapes sound perception. Previous research has shown that dance reflects the temporal structure of its accompanying music, communicating musical meter (i.e. a hierarchical organization of beats) via coordinated movement patterns that indicate where strong and weak beats occur. Experiments here investigated the effects of dance cues on meter perception, hypothesizing that dance could embody the musical meter, thereby shaping participant reaction times (RTs) to sound targets occurring at different metrical positions.In experiment 1, participants viewed a video with dance choreography indicating 4/4 meter (dance condition) or a series of color changes repeated in sequences of four to indicate 4/4 meter (picture condition). A sound track accompanied these videos and participants reacted to timbre targets at different metrical positions. Participants had the slowest RT’s at the strongest beats in the dance condition only. In experiment 2, participants viewed the choreography of the horse-riding dance from Psy’s “Gangnam Style” in order to examine how a familiar dance might affect meter perception. Moreover, participants in this experiment were divided into a group with experience dancing this choreography and a group without experience. Results again showed slower RTs to stronger metrical positions and the group with experience demonstrated a more refined perception of metrical hierarchy. Results likely stem from the temporally selective division of attention between auditory and visual domains. This study has implications for understanding: 1) the impact of splitting attention among different sensory modalities, and 2) the impact of embodiment, on perception of musical meter. Viewing dance may interfere with sound processing, particularly at critical metrical positions, but embodied familiarity with dance choreography may facilitate meter awareness. Results shed light on the processing of multimedia environments.

## Introduction

Because our understanding of the world is based on the integration of information that originates from different modalities, visual information is closely related to sound perception. Indeed, the interaction between auditory and visual information has been supported by numerous cross-modal studies [1–3]. For example, the McGurk effect clearly shows how individuals’ perception of a speech syllable changes when they watch a video of a mouth pronouncing other syllables [4]. The McGurk effect demonstrates that sounds are strongly associated with the movements by which the sounds are generated or with which they are accompanied. With the development of multimedia, music is often present in videos showing dancing or dance performances. Research on the effects of performers’ movements on music perception has shown that watching performers’ body movements changes the perception of various aspects of sound, such as music expressiveness [5], vibrato [6], note duration [7], phrasing [8], the size of sung intervals [9], and even the quality of the music performance [10, 11].

Although performers’ actions are the most common visual information accompanying music, dance is also a popular accompaniment. Many singers dance when they perform, and most music videos include dancers. Listeners may even dance while enjoying music. How, then, does dance actually affect individuals’ auditory perception of sound? Human body movement is closely related to the perception of rhythm [12, 13]. Previous experiments have shown that the manner in which people move their bodies to music affects their auditory perception of the rhythm structure [14]. In one experiment, adult participants were trained to bounce their knees in order to interpret the rhythm of an ambiguous rhythm as either a waltz (accent every three beats, triple meter) or a march (accent every two beats, duple meter). When tested later, participants perceived an auditory stimulus as having a duple or triple meter according to how they had moved their bodies previously, thereby demonstrating the impact of body movement on rhythm perception. This finding is also supported by a neuroscience study in which brain responses to rhythm were found to be significantly different when participants moved to the rhythm as opposed to simply hearing the rhythm [15]. Given that body movements shape the auditory perception of rhythm, we pose the following question: can simply watching body movements, such as dance movements, likewise influence rhythm perception?

The effect of visual stimuli on rhythm has mainly been studied by using a synchronization task in which people are asked to tap along to external isochronous visual beats. Previous studies have shown that synchronization to a visual metronome is poorer than that to an auditory metronome [16–18]. In addition, beat perception [19–21] and rhythmic interval timing tasks [22] have demonstrated the ineffectiveness of visual stimuli in the rhythm domain. However, the type of stimulus employed is important: studies with moving visual stimuli, such as bouncing balls and moving bars, lead to improved tapping synchronization [23–25] and rhythm perception [20]. A key component of the increase in rhythm perception is the spatiotemporal information provided by visually perceptible motion. A previous neuroscience study showed that synchronization to a moving visual stimulus activates the putamen, a brain region related to beat perception. Because human dance also includes periodic motions, viewing dance creates a visual rhythm, and this visual rhythm likely influences auditory rhythm perception. In addition, given research on mirror neurons that suggests that the mere visual observation of a goal-directed movement could elicit a neuronal representation and completion of the action [26, 27], solely watching a dance could improve rhythm perception as well. Indeed, a recent study [28] showed that the observation of simple movements enhances auditory rhythm perception. Specifically, a continuously bouncing human point-light figure, which was generated by recording a live human model bouncing the body regularly, improved the perception of and synchronization to auditory rhythm. However, the effect of observing more realistic and ecologically-valid dances on rhythm remains unclear. Therefore, the present study investigates how the viewing of dance videos affects concurrent sound perception.

In contrast to the simple bouncing of the knees, dance includes other periodic motions besides vertical movements upwards and downwards. The periodicity of a dance pattern is often closely connected to the temporal structure—the beat and meter—of the accompanying music. Beats are equally-spaced timing points that we hear as perceptually salient when listening to music. Beat induction makes it possible for us to tap to the pulse of the music. Meter refers to a hierarchical structure composed of those periodic beats or music regularities [29]. The hierarchical nature of meter causes some beats to be heard as stronger or relatively more salient. For example, a quadruple meter like 4/4 time (e.g. a march) causes a train of isochronous beats to be heard as a repeated cycle of four beats—strong, weak, medium, and weak beat—whereas a triple meter like 3/4 time causes a train of beats to be heard as a repeating cycle of three beats composed of a strong, weak, and weak beat (e.g. a waltz). Stronger beats are considered to occur on higher metrical levels [30]. Research on spontaneous dancing to music has shown that meter is embodied in dance with different metrical levels manifested by different components of the dance [31]. Thus, watching a dance, which often uses more elaborate motions compared to a simple bouncing motion, could elicit not only beat induction but also meter perception by observing the periodicities of dance motions. The aim of this study is to investigate meter perception as elicited by the observation of dance movements.

According to the dynamic attending model of rhythm perception [32–34], meter works as a real-time attention guide. Attention to meter facilitates the enhanced perceptual processing of information on beats with higher metrical levels (i.e. strong beats), as indicated by a wealth of research on meter and attention. For example, pitch accuracy judgments [34, 35] and just-noticeable temporal differences [36] are better for on-the-beat stimuli than for off-the-beat stimuli. Even visual discrimination [37] and word recognition [38] tasks are facilitated by metric sound sequences. In particular, Bolger et al. [39, 40] measured reaction times (RTs) to sound targets that occurred at four selected metrical positions. Participants listened to an isochronous auditory sequence in which an accent occurred every four beats; faster RTs were found for the accented targets (i.e. targets at higher metrical positions) compared to the unaccented targets (i.e. targets at lower metrical positions). By conducting the same sound detection task now accompanied by a dance video, we investigated how attention is guided by the viewing of dance motions.

In the first experiment, participants responded as quickly as possible to sound targets (timbre deviants). While the sound itself did not suggest any musical meter, the accompanying dance was designed to indicate a 4/4 time by repeating specific motions every four beats. By showing this dance video coordinated with the sound target detection task, we investigated whether watching dance could elicit the embodied meter of dance (4/4 time) and how this then affected sound perception. Modeled on the experimental task used in Bolger et al. [39], the timbre deviant targets appeared randomly at one of four selected metrical positions (MPs): the first beat (MP1), middle beat (MP5), the offbeat right before the first beat (MP8), and the offbeat right before the middle beat (MP4). In the metrical hierarchy specified by the dance, MP1 has the highest metrical position and MP5 has the next highest, while MP4 and MP8 occupy the lowest metrical positions. If the repetitive dance creates a sense of 4/4 time and guides attention to the metrically higher positions, the results should be the same as that of Bolger et al. [39]: RTs should be fastest for MP1 and slowest for MP4 and MP8 due to enhanced allocated attention to facilitate the processing of sounds at higher metrical positions. Alternatively, if the meter elicited by the dance causes attention to be allocated more to the visual domain, and therefore diverted away from the auditory domain, RTs to sound targets at MP1 would be the slowest due to reduced attention to the sounds (i.e. divided attention). Research on the connection between dance and music [41, 42] has found that music-dance synchrony gives rise to increased visual inspection times and greater attentional focus. Given that sound targets at MP1 have higher synchrony with the dance motion than targets at the other three metrical positions, participants might focus more visual attention to the dance and reduced attention to the auditory targets at MP1, resulting in slower RT’s. Previous experiments on multimodal attention have also shown that when attention is focused on a visual task, the auditory cortex decreases activity for irrelevant acoustic stimuli [43, 44]. In particular, tasks that require timed reactions to targets are more influenced by divided attention between different sensory modalities than untimed tasks [45]. Thus, it is possible that the limited resource of attention is shared for the processing of sound and dance, and therefore the timed sound detection task could be impacted by attention diverted to the visual domain to process the dance movements.

Finally, it is also possible that the observation of a dance does not induce a sense of meter but instead automatically captures attention by its salient features, such as those time points that feature the most physical motion. In this case, RTs to sound targets should be slower at the moments showing the largest motions, regardless of the meter. By quantifying the magnitude of motion for the dance at each metrical position where the target appeared, we also examined the relation between motion magnitude and the participants’ RTs.

To compare the bimodal effect of moving versus static visual stimuli on sound perception, we also added a picture condition in which the same sound detection task was performed while participants watched a circle at the center of the screen. By changing the color of the circle every four beats, we examined whether the color change could elicit a sense of the meter. Furthermore, the sound detection task with no accompanying video was conducted as a control condition. Through these three experimental conditions, we aimed to better understand how visual stimuli might shape sound perception, particularly in allowing listeners to perceive meter.

## Experiment 1

### Materials and Methods

#### Ethics Statement

**Fig 1:**
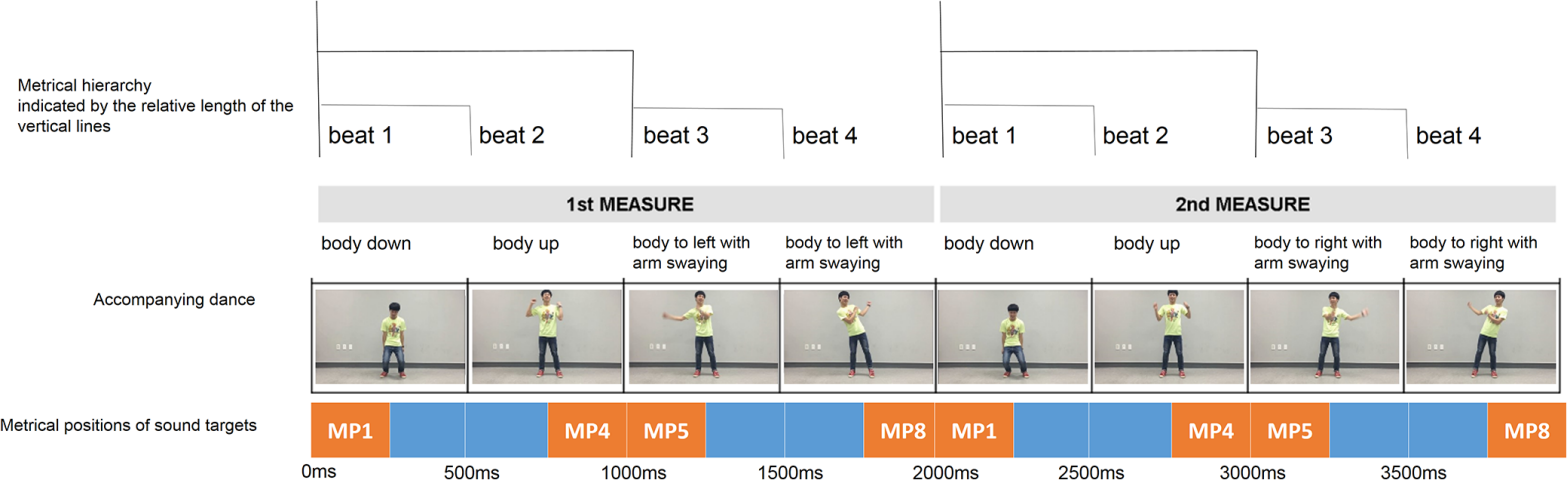
Schematic figure to show four metrical positions, where sound targets occur in reference to dance and the basic pattern of motions used in the dance condition. This pattern comprises two measures of four beats (one beat = 500ms), and it repeats 64 times for 256 seconds in a mini-block.

All participants gave written informed consent before participating in the experiment. The study was approved by the ethics commission of the Seoul National University Institutional Review Board and was conducted in accordance with the ethical standards of the 1964 Declaration of Helsinki. In addition, the individual dancing in the video shown to participants gave written informed consent (as outlined in the PLOS consent form) to publish the videos and related materials (Fig 1).

#### Participants

Twenty-three paid participants (9 females and 14 males; mean age = 21.5 years, SD = 2.04) took part in the experiment. The participants completed a questionnaire that assessed their music and dance experience in terms of age when training commenced, length of training, and type of training. Regarding musical expertise, most participants were non-musicians while 6 participants had roughly 10 years of formal musical training (Average years of musical training = 6.06 years, SD = 6.51). Regarding dance expertise, no participants were professional dancers. 2 participants had a few months of formal dance training (Dance training mean = 0.07 years, SD = 0.23).

#### Stimuli

*Auditory Stimuli*. The stimulus was a 256-second isochronous rhythm. Each tone was 50ms in length and was presented with an inter-onset interval (IOI) of 250ms. Based on a previous study [39] using the same stimulus, a beat lasted 500ms. The timbre of the tone was a “tick” click sound, and the pitch was D3 (146.83 Hz). The target was 100ms in length, and its pitch was F#6 (1479.98 Hz). A “ping” click was used for the timbre of the target (see S4 File). All sounds were generated using Audacity software (http://audacity.sourceforge.net).

*Visual Stimuli*. A newly-designed dance was used for the “dance” condition (Fig 1). To clearly indicate 4/4 time, the cycle of the dance motions exactly corresponded to the 4/4 meter. As Fig 1 shows, a dance pattern repeated every 2000ms, corresponding to one unit, or measure, of 4/4 time composed of the four beats (each beat of 500ms). One dance pattern consists of two parts: the first half includes the up and down movement of the body and arm, whereas the second half shows the arm swaying across the body from medial to lateral positions. The first and second half are further subdivided into two sub-components of 500ms, each corresponding to one beat. In addition, based on the results of previous research on embodied meter [31, 46], the dance movements were used to differentiate the metrical levels of beats (Fig 1). The mediolateral movements of the arms occurred every beat, while the lateral flexion of the upper torso occurred every two beats. To indicate the first beat of each measure (e.g. MP1), the whole body, including the arms, torso, and legs, was bounced only every four beats thereby visually accenting the downbeat. Overall then, the first beat (MP1) and the third beat (MP5) have larger motions than the other beats; moreover, MP1 is accented because of the whole-body movement while MP5 moves only limited parts of the body thus indicating a hierarchy of beats in keeping with 4/4 time. The dance was recorded and was paced by a sound sequence with an inter-beat interval of 500ms (120 BPM, see above description). Thus, the duration of one measure of the dance was 2 seconds (four 500ms beats). Since the dance pattern was performed alternately to the left and right side for 256 seconds, every odd measure was performed to the left side and every even measure was performed to the right side. The dance video was shown in a 22.5 cm wide and 14.5 cm high window (640×360 pixels) at 25 fps frames per second (see S1 File).

**Fig 2:**
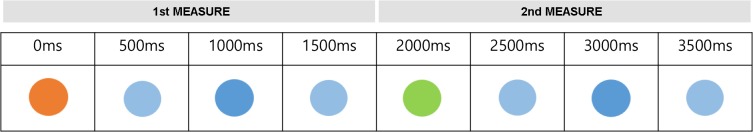
The basic pattern of the circle color changes used in the picture condition. This picture pattern comprises two measures of four beats (one beat = 500ms), and it repeats 64 times for 256 seconds in a mini-block.

For the “picture” condition, a circle 8 cm in diameter was centered on the monitor that changed color on every beat (i.e. every 500ms, see Fig 2). Orange was used for the first beat of every first measure, and yellowish green was used for the first beat of every second measure. Blue was used for the other three beats of each measure, but the third beat was visually accented by making it a slightly darker blue, while the second and fourth beat were lighter blue (Fig 2). Similar to the dance pattern, one picture pattern thus comprised two measures of four beats and repeated 64 times for 256 seconds. Within one measure, timbre sound targets on MP1 and MP5 were synchronized with the timing of the color changes, whereas the targets on MP4 and MP8 were not (see S2 File).

A “no video” condition was included as a control condition. In the no video condition, no visual stimuli were presented. Participants sat in front of the computer displaying a blank, white screen and only performed the auditory target detection task (i.e. audio only). Participants heard the isochronous rhythm track and responded to the timbre targets on the button box, which recorded their RTs.

#### Procedure

The task comprised of three blocks, one for each condition (dance, picture, and no video), with the order of presentation balanced across the participants. The participants were asked to respond to timbre targets as quickly as possible by pressing a button on a button box next to the computer. The independent variable manipulated in this experiment was the metrical position of the target presentation relative to the dance or picture. In keeping with the experiment by Bolger et al. [39], the target stimuli appeared at four chosen temporal positions: on the first beat of the dance (metrical position 1 or MP1), on the second and a half beat (metrical position 4 or MP4), on the third beat (metrical position 5 or MP5), or on the fourth and a half beat (metrical position 8 or MP8) as detailed above. Before the auditory stimulus was presented, one dance or picture pattern of eight beats was first played to visually prime a quadruple 4/4 meter. The first presentation of a target within a session could not occur before the first four seconds. Targets occurred randomly at the four metrical positions, but a target at the same metrical position could not occur consecutively, in order to diminish the possibility of a learning effect on RTs. The minimum duration between two consecutive targets was two seconds, so two consecutive targets could not appear within a single measure. Each block (dance, picture, and no video) lasted approximately 8 minutes, and each was subdivided into two mini-blocks of approximately 4 minutes. The participants were asked to attend to both the auditory and the visual stimuli during the experiment.

For all three experimental conditions, the participants were presented with 21 trials/metrical positions (total of 84 trials for each mini-block). The experiment was presented using E-prime software (Psychology Software Tools) for the stimulus presentation. The participants sat in front of a computer monitor, listened through Bose QuietComfort 25 Acoustic Noise-Cancelling headphones, and responded with the index or middle finger of their right hand on the button response box. Participants began each trial by pressing the space bar. The experiment lasted approximately 30 minutes in total. Before the main experiment, a practice session with 8 trials was provided to acquaint participants with use of the button box.

#### Motion Magnitude Analysis of Dance Videos

Because a digital video is comprised of a series of two-dimensional images, we can examine motion vectors through the transformations from adjacent frames in a video sequence. Commonly used strategies for motion estimation are block matching, hierarchical block matching, pel-recursive motion estimation, optical flow, and mesh matching [47]. In the present study, block matching was used to analyze the dance videos because it provides the most suitable standard estimation of motion magnitude. For details on the block-matching analysis (BMA), we referred to [47].

BMA is a standard technique for motion magnitude analysis due to its reliability and robustness with respect to encoding motions in video sequences [48]. The key idea behind BMA is to divide the current frame into a matrix of macro-blocks and to then compare those with the corresponding block and its adjacent neighbor macro-blocks in the previous frame to create a vector that specifies the movement of a particular macro-block in two adjoining frames. A displacement vector is estimated at a location (n_1_, n_2_, n) in the target frame. From there, template matching occurs, tracking the block centered on this point to respective blocks in a specified search area in the reference frame. We use the immediately prior frame as the reference [47]. The movement is calculated for all of the macro-blocks that compose a frame, and it constitutes the motion estimated in the current frame. The search area for a macro-block match is constrained up to p pixels on all sides of the corresponding macro-block in the previous frame. p is the search parameter and is generally 7 pixels.

After estimating all motion vectors in all frames, we calculated the magnitude of each vector in a frame. The cumulative sum of the magnitudes of each vector was used as a representative magnitude of the target frame. The basic pattern of the dance used for the first experiment was 4 seconds in duration, and the pattern repeated 64 times. Thus, the full length of the dance video was 256 seconds. The final motion magnitudes of the basic dance patterns were computed by averaging the values for every 4 seconds.

**Fig 3:**
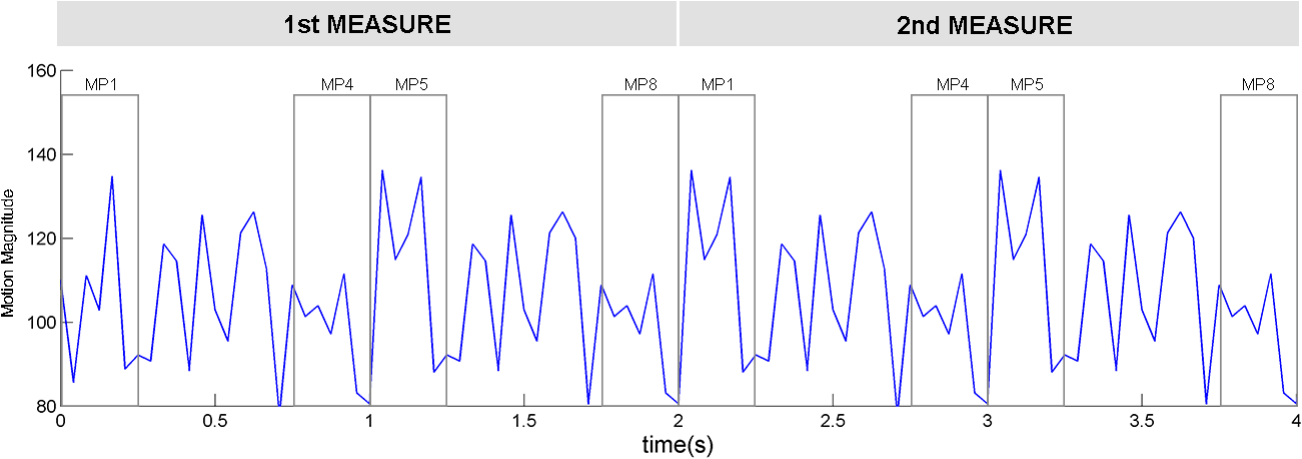
Motion magnitude changes in the dance pattern used for the first experiment displayed as a function of time. The basic dance pattern is 4 seconds, comprising two measures of four beats. The gray windows represent the timing points when the timbre targets of MP1, MP4, MP5, and MP8 are presented. The width of each window corresponds to the duration of each target. For the mean motion magnitudes used in Fig 4B, we averaged the magnitudes within the window of each metrical position for all measures.

The result of the motion magnitude analysis (Fig 3) shows that the newly-designed dance for the first experiment has very periodic magnitude changes occurring every one second, even though the motions of the first two beats and second two beats differ. The higher metrical positions, MP1 and MP5, have higher magnitudes than do the lower metrical positions, MP4 and MP8.

### Results

Reaction time (RT) data from participants were statistically analyzed via a 3 × 4 × 2 repeated-measures ANOVA to determine the effect of three within-subject factors: condition (dance, picture, control), metrical position (MP1, MP4, MP5, MP8), and measure (first, second). The assumption of normality was tested. Review of the S-W test for normality (*SW* = 0.995, *df* = 184, *p* = .831 for dance; *SW* = 0.987, *df* = 184, *p* = .078 for picture; *SW* = 0.987, *df* = 184, *p* = .095 for control) and skewness (0.065 for dance; 0.420 for picture; 0.352 for control) and kurtosis (0.187 for dance; 0.185 for picture; 0.020, for control) statistics suggested that normality was a reasonable assumption for our RT data. The results revealed a main effect of condition (*F*(2,44) = 12.48, partial η^2^ = .362, *p* = .000), with slower RTs in the dance condition than in the picture (Bonferroni, *p* = .012) and control (*p* = .000) conditions (dance, 381.74ms; picture, 365.22ms; control, 355.79ms), while RTs in the picture condition were not different from the control condition (*p* = .259). The main effect of metrical position (*F*(3,66) = 20.47, partial η^2^ = .482, *p* = .000) was significant, with the slowest RTs at MP1 (Bonferroni, *p* = .000 for MP4, *p* = .002 for MP5, *p* = .000 for MP8; MP1, 375.90ms; MP4, 364.41ms; MP5, 368.35ms; MP8, 361.66ms). The main effect of measure was also significant (*F*(1,22) = 6.30, partial η^2^ = 0.223, *p* = .020), with faster RTs in the first measure than in the second measure (Bonferroni, *p* = .020; first, 366.20ms; second, 368.96ms). The interaction of condition × metrical position × measure was not significant. Measure did not yield an interaction with any of the two other factors.

**Fig 4:**
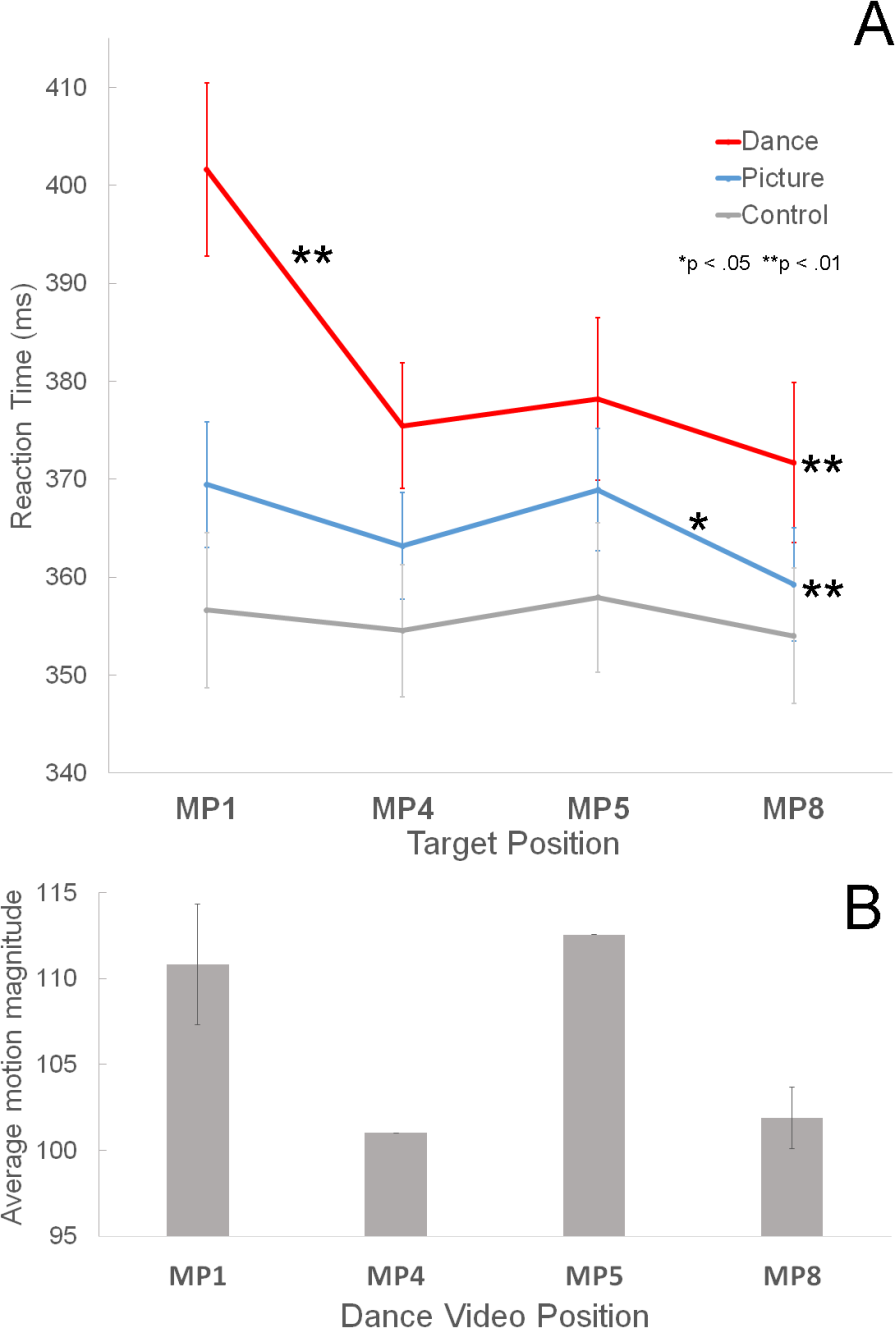
Results of Experiment 1. **A.** The mean RTs for the four metrical positions for each condition when averaged across measure. Error bars represent ± SE. * *p* < 0.05, ** *p* < 0.01 **B.** The mean motion magnitude of the dance video corresponding to each metrical position. Error bars represent ± SD.

Most importantly, the interaction of condition × metrical position was significant (*F*(6,132) = 9.00, partial η^2^ = .290, *p* = .000) indicating different reaction times to the various metrical positions within the different experimental conditions (Fig 4A). Table 1 shows the mean RTs for the four metrical positions for each condition when averaged across measure. *Post-hoc* tests using the Bonferroni correction revealed that RTs significantly differed depending on the metrical position for the dance and picture conditions, but not for the control condition. Specifically, in the dance condition, RT for the targets at MP1 were significantly slower from RT’s for the other metrical positions (*p* = .000 for MP4, MP5 and MP8), while those for the other three MPs were not. Thus, in the dance condition, the strongest metrical position, MP1, elicited the slowest reaction times. In the picture condition, only RTs for MP8 were significantly different than those for MP1 (*p* = .000) and MP5 (*p* = .019), while RTs for other MPs did not differ. In the control condition, RTs for all four metrical positions were not significantly different.

**Table 1:** Average RT and standard deviation (ms) for each metrical position of each condition.

Condition	MP1	MP4	MP5	MP8
Dance	401.59 (42.36)	375.47 (30.91)	378.18 (39.80)	371.70 (39.14)
Picture	369.48 (30.76)	363.20 (26.01)	368.94 (29.97)	359.26 (27.93)
Control	356.64 (38.03)	354.55 (32.38)	357.93 (36.64)	354.02 (33.05)

Fig 4B shows the average (mean) motion magnitudes when each target was presented. MP1 and MP5 had larger motion magnitudes than MP4 and MP8 (Fig 4B), but RTs were significantly different only for MP1 within both measures. Thus, motion magnitude is not directly related to the RTs.

In summary, the dance condition showed slower RTs than the picture and control conditions, whereas the picture condition did not differ from the control condition. The second measure showed faster RTs than the first. The interaction of condition and metrical position indicated that RTs for MP1 were significantly slower only in the dance condition. In the picture condition, RTs for MP8 were significantly faster. However, in the control condition, there were no RT differences among the MPs.

## Experiment 2

In the first experiment, the dance condition used a newly-designed dance, and RTs were significantly different only for MP1. The novelty of the dance may have caused the dance video to have a weaker impact on meter perception compared to a dance video that is more familiar to our participants. To examine this, in the second experiment, we conducted the same target detection task with the well-known choreography of the horse-riding dance from the popular song “Gangnam Style” by pop-singer Psy [49]. Unlike the newly-designed dance in Experiment 1, the horse-riding dance is associated with a specific song, “Gangnam Style” and participants may have experience with the motions of this dance. The associated memory and experience with this choreography and music may cause participants to deduce a clearer and more detailed sense of meter when watching the horse-riding dance video. To examine this possible impact of familiarity, we compared the responses of two subject groups, participants with and without experience performing the horse-riding dance. Via a questionnaire that we administered, all participants responded that they had seen the horse-riding dance before, so it was predicted that viewing the dance would lead to greater RT differences among the metrical positions compared to the results seen for the first experiment. Additionally, by dividing the participants into two groups—those who had performed the horse-riding dance and those who had not—we were able to examine the effect of embodied experience with the dance on auditory RTs. Given the effect of direct body movement on rhythm perception observed in previous studies [12–14], we hypothesized that this dance video would have a more significant effect on the participants’ RTs compared to the results in Experiment 1 and RT differences would exist between the two subject groups.

### Ethics Statement

**Fig 5:**
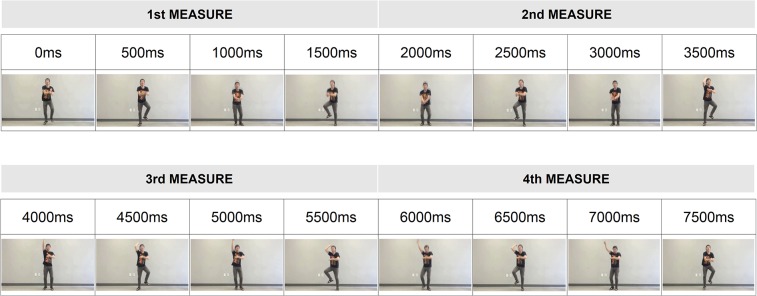
The basic dance pattern of the horse-riding dance video used for the second experiment. The dance pattern comprises four measures of four beats (one beat = 500ms), and it repeats 32 times for 256 seconds in a mini-block.

All participants gave written informed consent before participation in the experiment. The study was approved by the ethics commission of the Seoul National University Institutional Review Board and was conducted in accordance with the ethical standards of the 1964 Declaration of Helsinki. The individual dancing in the video shown to participants gave written informed consent (as outlined in the PLOS consent form) to publish the videos and related materials (Fig 5).

### Participants

27 paid participants took part in this experiment (7 female and 20 male; mean age, 22.5). 19 of 27 participants performed in both Experiment 1 and 2, and for these participants, the order of experiments was counterbalanced. Most participants were non-musicians, but six of them had about 10 years of musical training. No participants had professional dance training. On the administered questionnaire, all 27 participants responded that they had seen the horse-riding dance to the song “Gangnam Style” at least once before. Furthermore, 16 participants reported experience dancing the horse-riding dance more than once before, and 11 reported that they did not.

### Stimuli

The horse-riding dance from “Gangnam Style” was shown for the target detection task that was used in the first experiment. The original horse-riding dance in the music video by Psy (https://youtube/9bZkp7q19f0) occurs at a tempo of 134 BPM (beat per measure). The video for this experiment recorded by our dancer was paced to a sound sequence with an inter-beat interval of 500ms (120 BPM) in order to achieve a dance tempo similar to the first experiment. The horse-riding dance included a bouncing movement of the body every beat, along with a jumping pattern of the legs (see S3 File).

The change in motion direction and the rise or fall of the right arm indicated the different metrical levels. The highest level described by one dance pattern consisted of four measures of four beats (total 16 beats, 8 seconds), in which the two-measure pattern (8 beats, 4 seconds) repeated two times, once without and once with the right arm raised high (see S3 File for video of the dance). Within the two-measure unit, the two-second motion repeated, first with the leg bouncing to the right and then with the leg bouncing to the left. The four-measure dance repeated for 256 seconds (Fig 5).

### Procedure

The experimental task and environment were identical to those used in the dance condition in the first experiment. Participants only participated in a dance condition in Experiment 2.

### Motion Magnitude Analysis

**Fig 6:**
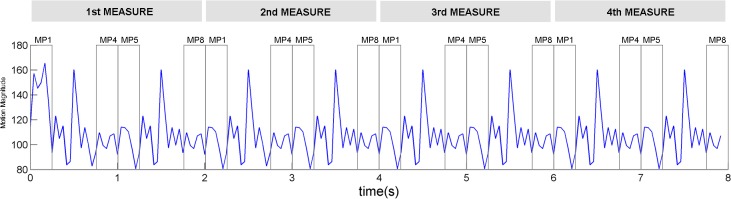
Motion magnitude changes in the horse-riding dance pattern used for the second experiment as a function of time. The basic dance pattern is 8 seconds, and it comprises four measures of four beats. The gray windows represent the timing points when the sound targets of MP1, MP4, MP5, and MP8 are presented. The width of each window corresponds to the duration of each target.

The basic pattern of the horse-riding dance was 8 seconds, and the pattern repeated 32 times for the second experiment. Thus, the full length of the video was 256 seconds. The final motion magnitudes of the basic dance pattern were computed by averaging the values for every 8 seconds for the second experiment. The results of the motion magnitude analysis for the horse-riding dance are illustrated in Fig 6. Compared to the dance in Experiment 1, most MPs in Experiment 2 have relatively small motion magnitudes. Even MP1 and MP5, MP’s occurring at higher metrical levels, do not have large motion magnitudes. In fact, MP1 of the first measure is the only MP with a large motion magnitude due to the motion of the right arm.

### Results

A repeated-measures ANOVA was conducted to identify the effects of two within-subject factors: metrical position (MP1, MP4, MP5, MP8) and measure (first, second), and one between-subject factor, group (with and without horse-riding dance experience), on the RT results. For the measure factor, we compared every first and second measure, similar to the analysis in first experiment. That is, due to the repetition of the horse-riding dance pattern (Fig 5), every first and third measure (i.e. odd measures) was considered as the first measure, and every second and fourth measure (i.e. even measures) was considered as the second measure. This categorization was due to the fact that the same dance movements occurred but angled in different directions; the legs bounced to the right in the odd measures and to the left in the even measures. This approach was used because an ANOVA comparison of the first two and second two measures to each other did not show a significant main effect of measure. The assumption of normality was tested. Review of the S-W test for normality (*SW* = 0.980, *df* = 128, *p* = .061 for the group with horse-riding dance experience; *SW* = 0.984, *df* = 80, *p* = .418 for the group without experience) and skewness (0.309 for the group with horse-riding dance experience; 0.144 for the group without experience) and kurtosis (0.162 for the group with horse-riding dance experience; -0.341 for the group without experience) statistics suggested that normality was a reasonable assumption.

The results showed a significant effect of metrical position on the RTs (*F*(3,75) = 23.48, partial η^2^ = .484, *p* = .000). Unlike in Experiment 1, RTs for MP1 were not significantly different from MP4 and MP5, but they were significantly slower than RT’s at MP8 (Bonferroni, *p* = .77 for MP4; *p* = .085 for MP5; *p* = 0.000 for MP8; MP1, 377.07ms; MP4, 368.10ms; MP5, 384.08ms; MP8, 360.42ms). RTs for MP5 were significantly slower than RTs for MP4 and MP8, but not from MP1 (Bonferroni, *p* = .000 for both MP4 and MP8, *p* = .085 for MP1). RTs for MP8 were significantly faster than RTs for the other three MPs (Bonferroni, *p* = .000 for both MP1 and MP5, *p* = .048 for MP4), while MP4 showed significant differences only from MP5 and MP8 (Bonferroni, *p* = .077 for MP1, *p* = .000 for MP5, *p* = .048 for MP8). Thus, in general, MP1 showed relatively slow RTs, while MP8 showed fast RTs. The effect of measure was significant (*F*(1,25) = 8.52, partial η^2^ = .254, *p* = .007), showing significantly faster RTs (*p* = .007) for the second measure (369.33ms) compared to the first (375.50ms) measure. There was no significant effect of group (*F*(1,25) = 1.39, *p* = .250).

**Fig 7:**
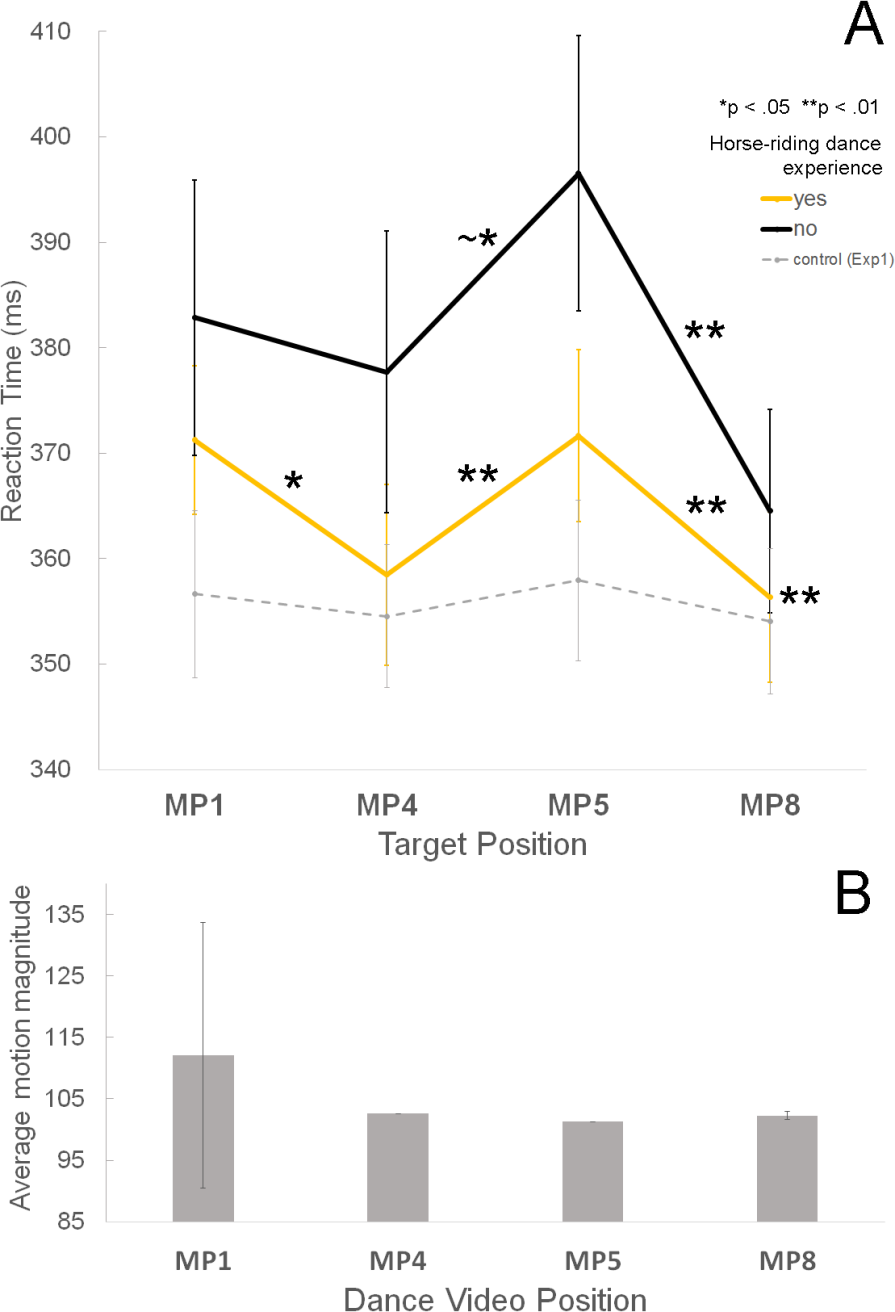
Results of Experiment 2. **A.** The mean RTs for the four metrical positions for each group, with experience (yellow line) and without experience (black line) with the horse-riding dance. The dotted gray line represents the mean RTs in the control condition of Experiment 1. Error bars represent ± SE. * *p* < 0.05, and ** *p* < 0.01. ~* indicates marginal significance at the level of p = 0.05 **B.** The mean motion magnitude derived from the dance video for each metrical position. Error bars represent ± SD.

The metrical position × group interaction was significant indicating that the two subject groups behaved differently at the different metrical positions (*F*(1.96, 48.93) = 4.29, partial η^2^ = 0.146, *p* = .020, see Fig 7 and Table 2). For this interaction, Mauchly’s test indicated that the assumptions of sphericity had been violated, and degrees of freedom were therefore corrected using Greenhouse-Geisser estimates of sphericity. *Post-hoc* tests using the Bonferroni correction revealed that for the group without experience with the horse-riding dance, only RTs for MP5 showed significant or marginally significant differences from all the other MPs (*p* = .022 for MP1, *p* = .052 for MP4, *p* = .001 for MP8; MP1, 382.88ms; MP4, 377.71ms; MP5, 396.51ms; MP8, 364.52ms), whereas the other three MPs did not.

**Table 2:** Average RT and standard deviation (ms) for each metrical position in each group.

Horse-riding dance experience	MP1	MP4	MP5	MP8
Yes	371.26 (28.25)	358.49 (34.33)	371.65 (32.68)	365.09 (34.27)
No	382.88 (43.30)	377.71 (44.39)	396.51 (43.28)	364.52 (32.01)

By contrast, for the group with experience, MP5 showed significantly slower RTs from RTs for MP4 and MP8, but not from MP1 (*p* = 1.000 for MP1, *p* = .004 for MP4, *p* = .002 for MP8; MP1, 371.26ms; MP4, 358.49ms; MP5, 371.65ms; MP8, 356.33ms). RTs for MP1 were also significantly slower from those for MP4 and MP8, but not from MP5 (*p* = .049 for MP4, *p* = 1.000 for MP5, *p* = .005 for MP8). RTs for MP4 differed from those for MP1 and MP5, but not from MP8 (*p* = .049 for MP1, *p* = .004 for MP5, *p* = 1.000 for MP8).

In Fig 7B, the displayed mean motion magnitude shows magnitude averaged across all measures in the dance video. The motion magnitude was larger only for MP1, but this is mostly because of MP1 of every first measure where the right arm was moved. Fig 6 shows that MP1s of other measures do not have larger motion magnitude averages. Although MP1 shows the largest motion magnitude, the participants’ RTs were significantly different between MP1 and MP5, indicating that the motion magnitude is not directly related to RTs.

**Fig 8:**
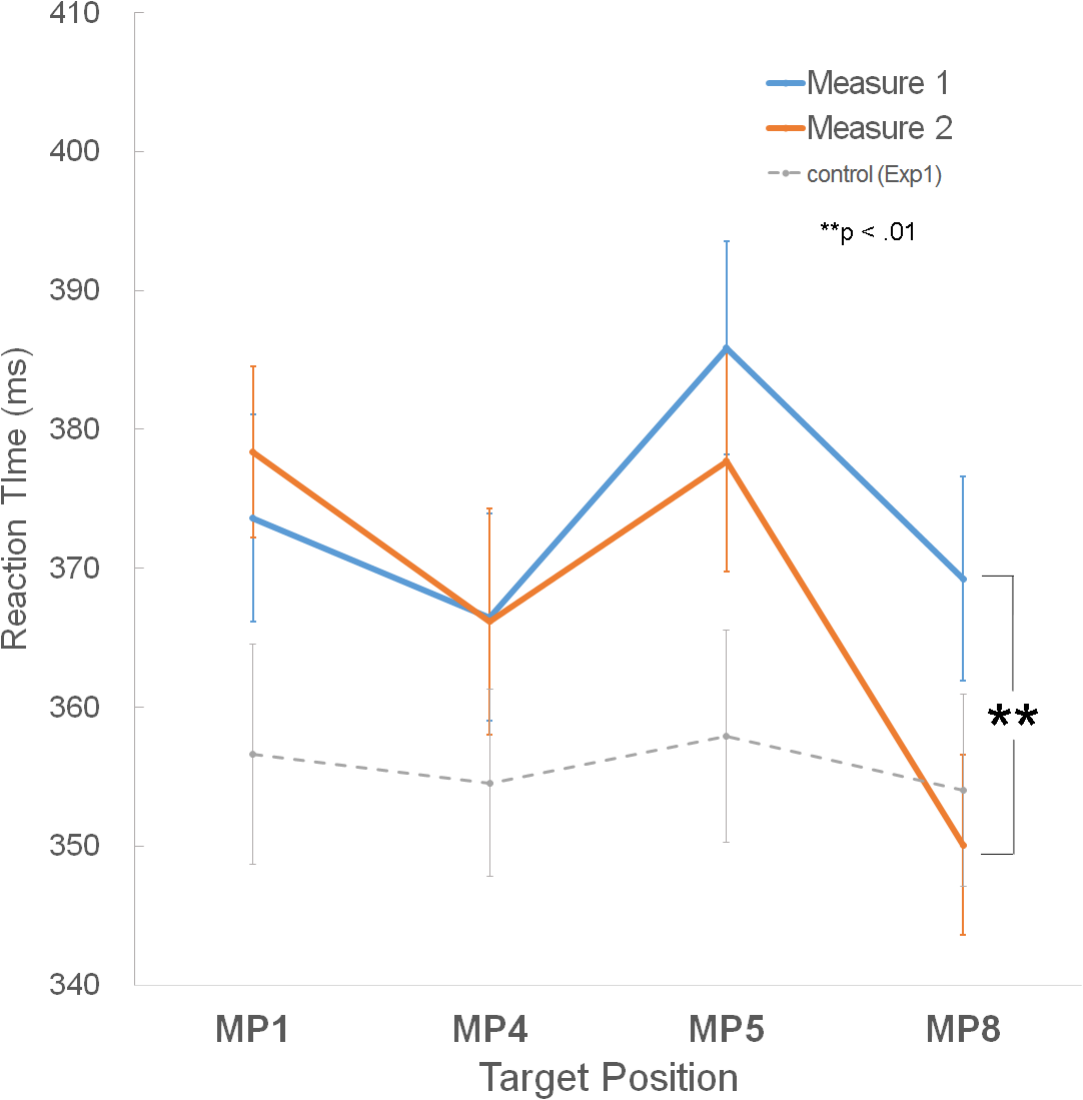
Results of Experiment 2. **A.** The mean RTs for the four metrical positions in the first (blue line) and second measure (orange line). The gray dotted line represents the mean RTs in the control condition of Experiment 1. Error bars represent ± SE. ** *p* < 0.01.

The interaction of the metrical position x measure was significant (Fig 8 and Table 3). Only MP8 showed significant RT differences between measure 1 and 2 (*p* = .007; measure 1, 369.25ms; measure 2, 350.08ms). This result indicates that the RTs differences between the two measures are mostly due to the RTs at MP8. The interaction of measure × group (*F*(1,25) = 1.40, *p* = .248) and the interaction of measure × metrical position × group (*F*(3,75) = 0.04, *p* = .988) were not significant.

**Table 3:** Average RT and standard deviation (ms) for each metrical position of each measure.

Measure	MP1	MP4	MP5	MP8
First	373.62 (38.87)	366.45 (38.73)	385.85 (39.76)	369.25 (38.29)
Second	378.37 (32.09)	366.18 (42.26)	377.71 (41.15)	350.08 (33.70)

In summary, the results for experiment 2 showed that in general, the stronger metrical positions, MP1 and MP5, have slower RTs than the weaker metrical positions, MP4 and MP8, and that the second measure showed faster RTs than the first measure. Regarding group comparisons, the group without experience with the horse-riding dance showed slower RTs to MP5 than to the other three MPs, whereas for the group with experience, RT’s to both of the stronger metrical positions, MP1 and MP5, were slower than RTs to MP4 and MP8. The significant interaction of measure and MPs indicates that only MP8 of the second measure showed slower RTs than that of the first measure.

## Discussion

The present study examined whether the visual input provided by periodically-repeating dance motions and picture color changes elicit a sense of meter. Specifically, the two experiments in this study examined whether the elicited meter influenced sound detection by measuring participants’ RTs to deviant sounds at four metrical positions with different metrical levels as indicated by the visual dance or picture stimuli. This study was modeled after a previous study by Bolger et al. [39], where participants performed the same sound detection task while simultaneously listening to a rhythmic entrainment sound indicating a 4/4 meter. In our experiment, by using the same auditory task as in Bolger et al. [39] but now accompanied by a dance video instead of sound alone, we aimed to explore the role of dance viewing on sound perception.

Our experimental results indicated that RTs were significantly slower in the dance condition than in the picture and control condition. In contrast to the study by Bolger et al., which found the fastest RTs for the highest metrical position, MP1 (the first beat of a measure), our study found the slowest RTs for MP1 in the dance condition. The second experiment in our study, which used the familiar horse-riding dance from “Gangnam Style,” revealed a significant group and metrical position interaction. That is, the group with experience with the choreography of the horse-riding dance showed significantly slower RTs for the targets at both MP1 and MP5 than for the targets at MP4 and MP8. The interaction of measure and MPs were also significant, demonstrating faster RTs for MP8 only in the second measure. The implications of these different results are discussed below.

### Slower RTs for the Highest Metrical Position only in the Dance Condition

The finding of slower RTs for the dance condition than for the picture and control condition suggests that the presence of the dance video itself generally increased participants’ perceptual load [50]. However, the impact of the video on auditory perception differed depending on the metrical position. While previous experiments found that listening to a 4/4-time rhythm sequence reduced the RTs for sound targets at MP1, the highest metrical pulse level [39] in accordance with the dynamic attending model, viewing the dance video embodying a 4/4 time lengthened RTs for MP1 in our Experiment 1. While several experiments have found privileged perceptual processing at strong metrical positions as summarized in the introduction, this may be dependent on the particular context. For example, research on real-time attention to multi-voiced canons (i.e. compositions where two or more parts sing or play the same music starting at different times) also found slower RT’s at strong metrical positions. This was likely due to the overlapping of phrases in the canons; because one phrase began as the other one ended, this may have weakened the metrical stability of the downbeat [51]. Our experimental results here may be due to the fact that visual information, in the form of a dance video, appears to guide attention in a different manner than if auditory information is processed alone. Slow RTs to sound targets at MP1 might indicate that more attention was allocated to the visual domain (i.e. towards the dance) at MP1 and relatively reduced attention to the auditory targets, decreasing the efficiency of auditory processing at MP1. That is, when we listen to sounds along with an accompanying dance video, vision and hearing share limited attentional resources; the embodied meter of dance guides attention to the dance at metrically higher levels, resulting in the decreased efficiency of sound processing. Attention is divided. As mentioned in the introduction, previous studies on multimodal attention have shown that when attention is focused on a visual task, the auditory cortex decreases activity for irrelevant acoustic stimuli [43, 44]. In particular, tasks that require timed reactions to targets are more influenced by dividing attention between different sensory modalities than are untimed tasks [45].

It is not likely that the momentary visual saliency caused by the high degree of motion magnitude at MP1 automatically captures visual attention. The BMA in this study showed that MP1 and MP5 have the same degree of motion magnitude but that RTs were significantly slower only for MP1 in the first experiment. This result lends credence to the idea that the internal meter elicited by the repeated dance pattern, not the visual saliency of the dance itself, guides attention to higher metrical positions.

Unlike Experiment 1, results from Experiment 2 showed the slowest RTs at MP5 (Table 4). This discrepancy could be explained by the difference in the dances used for the two experiments. The dance used in Experiment 1 emphasizes the MP1 metrical position and the dance used in Experiment 2 emphasizes the MP5 metrical position as the strongest beat. The dance for Experiment 1 includes the movement of the whole body going downward only at MP1. Since downward motion is closely related to the embodiment of beat [31], MP1 is visually accented by the dance in Experiment 1. In the dance for Experiment 2, however, MP1 visually looks similar to the other metrical positions. In the horse-riding dance, MP5 is the metrical position that is visually accented due to the abrupt change in leg movement pattern. Specifically, the dancer usually changes the stomping leg between right and left every beat, but he suddenly stops this change at MP5. This sudden change of the dance pattern makes MP5 perceptually salient, causing attention to switch from the auditory to the visual domain. Thus, “Gangnam style” emphasizes the third beat as the highest metrical level overall; this is reflected both in the accompanying dance and by the lyrics, since the titular phrase ‘Gangnam style’ also appears every third beat of each measure. Thus MP5 is made salient by the combination of auditory and visual cues, and this is reflected in participants’ slower reaction times at MP5.

**Table 4:** Average RT and standard deviation (ms) for each metrical position of Experiment 1 and 2.

	MP1	MP4	MP5	MP8
Exp1	401.59 (42.36)	375.47 (30.91)	378.18 (39.80)	371.70 (39.14)
Exp2	375.99 (34.86)	366.32 (39.12)	381.78 (38.63)	359.66 (31.85)

In both Experiment 1 and 2, RTs were not finely discriminated among all metrical positions. Only one metrical position, the strongest metrical position (MP1 for Experiment 1 and MP5 for Experiment 2), elicited significantly slower RTs. These results suggest that there is a limit, or threshold, to the metrical levels demarcated by the dances used in the experiments. While the highest metrical position is marked well in the dances for the two experiments, the other positions are not. Alternatively, it is also possible that the various levels of meter are adequately reflected in the dance to discriminate at all metrical levels, but the meter was not able to direct or sculpt the fine allocation of attention. Further studies are needed to examine this issue.

Interestingly, it appears that familiarity, or experience, can allow participants to more finely discriminate and perceive the metrical hierarchy. In Experiment 2, the group with experience performing the horse-riding dance choreography showed more discrete RT differences between the strong (MP1 and MP5) and the weak (MP4 and MP8) metrical positions. That is, the group with experience showed slower RTs for both MP1 and MP5 (strong metrical positions) than MP4 and MP8 (weak metrical positions), whereas the group without experience showed slower RTs only for MP5. This result indicates that physical experience with performing the motions of a dance (i.e. embodiment) helps individuals allocate attention to audiovisual stimuli in a more refined and specific way. Previous studies have demonstrated the effects of body movements on rhythm perception [14, 15], and the present study lends support to the idea that familiarity with body movements contributes to an enhanced discrimination of different metrical levels.

### Non-significant Effects of Picture on Sound Detection

Compared to the dance condition, RTs in the picture condition, our experimental condition that used static visual changes, did not differ from the control condition. This result indicates that visual stimuli that lack spatiotemporal information have no effect on simultaneous sound perception. This result is consistent with previous studies illustrating the weak influence of visual flickers on rhythm [16, 52–54]. Furthermore, the picture condition did not show significant RT differences for metrical positions occurring at higher metrical levels. Although MP1 and MP5 exhibit higher temporal synchrony between color changes and sound deviants than MP4 and MP8, this difference was not reflected in the participants’ RTs. Instead, MP8 showed significantly faster RTs than the other metrical positions. These relatively fast RTs at MP8 may reflect the recognition of a repeated picture pattern. Although simple visual stimuli do not seem to elicit a strong sense of meter, participants may be aware of the repeating color sequence pattern used here. Thus, participants might have developed expectations, predicting that the visual pattern would end soon after they saw the last circle color of the pattern. This awareness and pattern expectation might cause participants to shift attention from the visual domain back to the auditory domain, as it was no longer necessary to focus intensely on the visual pattern, resulting in the faster RTs to sound targets at MP8. That is, the later a sound target occurs within the repeating visual pattern, the faster the RTs to that target. This behavior is also consistent with the findings in Experiment 2, where RTs were the fastest at MP8 of the second measure, not in the first measure; participants were habituated to the visual pattern and could therefore shift attention away from the visual domain to focus on the auditory domain, again suggesting a possible effect of expectation due to pattern recognition.

## Conclusion

By measuring RTs to sound targets at different metrical positions when participants observed accompanying dance motions and picture patterns, this study explored the impact of viewing dance on simultaneous sound perception. Since the body movements of dance are synchronized with the temporal structure of the accompanying music, meter may be induced simply by watching periodic dance movements. The finding of slower RTs for sound deviant targets at higher metrical positions of dance suggest that: 1) the viewing of dance orients attention to the visual domain at metrically higher positions indicated by the embodied meter of the dance movements and that 2) this divided attention between visual and auditory domains selectively decreases the efficiency of auditory processing because of the higher perceptual load. In the second experiment of this study, RT differences between high and low metrical positions were more salient in the group with experience performing the horse-riding dance choreography than in the group without such experience. These results suggest that physical experience and familiarity with performing a dance, where motions are embodied and internalized, enhances selective discrimination of metrical positions.

## Supporting Information

S1 File The dance video used for the dance condition of Experiment 1. (MP4)

S2 File The picture video used for the picture condition of Experiment 1. (MP4)

S3 File The dance video used for the dance condition of Experiment 2. (MP4)

S4 File An example of auditory stimuli used for blocks of Experiment 1 and 2. (WAV)
